# Endoscopic stent placement above the sphincter of Oddi for biliary strictures after living donor liver transplantation

**DOI:** 10.1186/s12876-020-01226-x

**Published:** 2020-04-06

**Authors:** Mitsuhito Koizumi, Teru Kumagi, Taira Kuroda, Yoshiki Imamura, Kozue Kanemitsu, Kohei Ogawa, Yasutsugu Takada, Yoichi Hiasa

**Affiliations:** 1 Gastroenterology and Metabology, Ehime University Graduate School of Medicine, Shitsukawa, Toon, Ehime 791-0295 Japan; 2 Hepato-Biliary-Pancreatic Surgery and Breast Surgery, Ehime University Graduate School of Medicine, Toon, Ehime Japan

**Keywords:** Endoscopic biliary drainage, Living donor liver transplantation, Biliary stricture

## Abstract

**Background:**

Endoscopic balloon dilation and/or plastic stent placement has been a standard method for treating biliary strictures complicated post living donor liver transplantation (LDLT). The strictures may be refractory to endoscopic treatment and require long-term stent placement. However, consensus on the optimal period of the stent indwelling and usefulness of the inside stent does not exist.

**Methods:**

We evaluated the long-term efficacy of stent treatment in patients with biliary stricture post LDLT. In addition, we compared the stent patency between inside stent and conventional outside stent.

**Results:**

A total of 98 ERC sessions (median 6: range 1–14) performed on 16 patients receiving endoscopic treatment for biliary strictures post LDLT with duct-to-duct biliary reconstruction were analyzed. Biliary strictures successfully treated in 14 patients (88%) included 7 patients (44%) showing improvement of biliary strictures with repeated endoscopic stent placement. Stent replacement was carried out every 6 to 12 months for the remainder 7 patients (44%). Biliary stents were placed in 87 sessions (77 inside sessions and 10 outside sessions). Stent migration occurred 13 times (16%) and none of the inside stent sessions and the outside stent sessions, respectively. Median patency of inside stent and outside stent were 222 days (range; 8–1736) and 99 days (range; 7–356), respectively. The stent occlusion was significantly less in inside stent than in outside stent (*p* < 0.001). Stone formation was observed in 14 (18%) of the inside stent and 3 (30%) of the outside stent. Biliary stones were small and successfully removed endoscopically.

**Conclusions:**

The endoscopic treatment using inside stent was useful in the management of biliary strictures after LDLT.

## Background

In Japan, living donor liver transplantation (LDLT) is more common than deceased donor liver transplantation (DDLT) due to a shortage of deceased donor organs [1, 2]. Biliary strictures after LDLT are common complication counting for 15–30%, despite advances in surgical techniques, organ preservation and immunosuppressive management [3, 4]. Endoscopic balloon dilation and/or plastic stent placement had been a standard method to treat biliary strictures after liver transplantation with duct-to-duct biliary reconstruction [5–7]. In some cases, however, the strictures may be refractory to therapeutic endoscopy and may require long-term stent placement or surgical revision from the duct-to-duct anastomosis to the hepaticojejunal anastomosis [8, 9]. A major issue of long-term stent placement is the requirement for periodic stent replacement due to insufficient patency period of plastic stents. Generally, the stent is located across the papilla and its distal end is exposed to the duodenum. This may lead to free reflux of duodenal contents through the stent, which is believed to be the major cause of stent occlusion [10, 11]. Therefore, plastic stent usually requires prophylactic replacement every 2 to 4 months, especially in immunocompromised LDLT patients [12]. To avoid this complication and to aid prolongation of stent patency, usefulness of inside stent placed into proximal to the sphincter of Oddi has been reported [13, 14].

Although there are a few studies showing treatments for benign bile duct stricture, an optimal period of stent indwelling and usefulness of inside stent has not obtained a consensus. In our study, we retrospectively evaluated the safety and long-term efficacy of stent treatment in patients with biliary stricture after LDLT. Furthermore, we compared the stent patency between inside stent placement and conventional outside stent placement.

## Methods

### Patient selection

Sixteen consecutive patients with a median age of 55.5 years (range, 36–67 years) suffering from biliary strictures after LDLT with duct-to-duct biliary reconstruction for end-stage liver disease or acute liver failure at our hospital between 2004 and 2016 were eligible. Anastomotic biliary strictures were diagnosed by endoscopic retrograde cholangiography (ERC). Patients with non-anastomotic biliary strictures were excluded.

### Endoscopic treatment

**Fig. 1:**
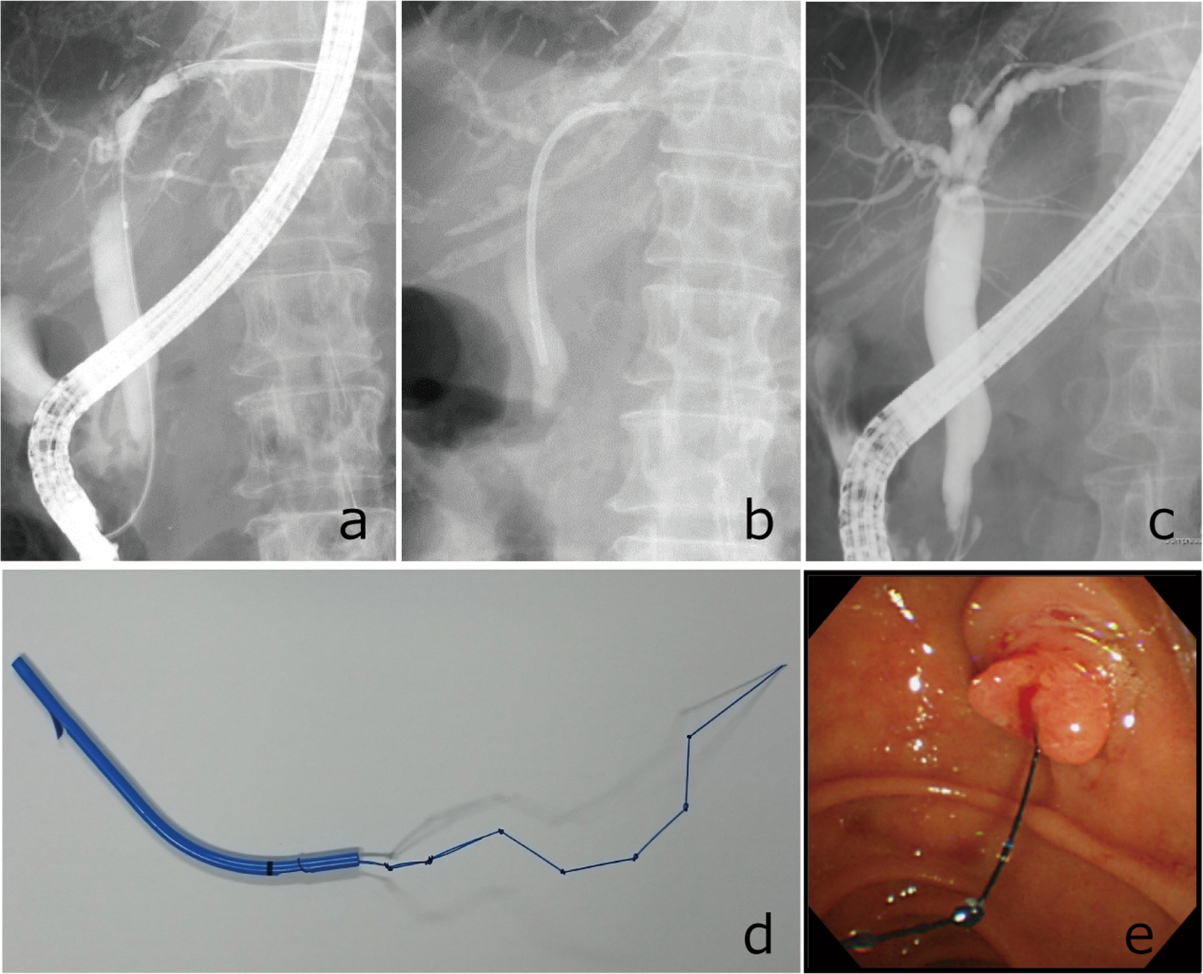
Procedure of endoscopic treatment for biliary stricture. **a** Cholangiogram shows a biliary stricture. **b** Placement of an inside stent across the biliary stricture above the sphincter of Oddi. **c** Cholangiogram shows the resolution of the stricture after exchanging the inside stent. **d** Whole view of an inside stent. **e** Duodenal papilla with inside stent placed

Informed consent for the treatment was obtained from patients and their families prior to endoscopic treatment. ERC was performed under conscious sedation, using duodenoscopes (JF-260 V, TJF-260 V, Olympus Corporation, Tokyo, Japan) (Fig. 1a). The cholangiographic findings were classified according to the shape of the stricture site and the shape of the distal duct, as previously reported [15]. After confirming the stricture with cholangiography, guide wires (VisiGlide, Olympus Corporation, Tokyo. Radifocus, Terumo, Tokyo, Japan) were used to pass the strictures.

Outside stents were placed across the sphincter of Oddi, with their distal end exposed into the duodenum. Inside stents being placed above the sphincter of Oddi (Fig. 1b, e). Endoscopic sphincterotomy (EST) was not performed for both methods at initial attempt. The shape of a 7-10Fr plastic stent (Flexima; Boston Scientific Japan, Tokyo, Japan. Cotton-Leung Sof-Flex Biliary Stent; Cook Japan, Japan. ThroughPass; Gadelius Medical, Tokyo, Japan) was modified as an inside stent. The distal flap of the stent was removed but instead a nylon thread was attached to the distal side hole beyond the duodenal papilla to facilitate retrieval of the inside stent, as previously reported (Fig. 1d) [16]. The number and length of the stent were selected depending on the location and types of strictures. Either outside stent or inside stent was selected according to preference of board-certified endoscopists. In more recent years, inside stent was inserted preferentially in our institution.

### Evaluation of stent patency and biliary stricture

Patient follow up after stenting was carried out by abdominal X-rays and liver profiles (bilirubin and hepatobiliary enzymes) every 1–2 months. ERC sessions were scheduled every 6 to 12 months regardless of symptoms or abnormalities in liver profiles. At the next session, the stent was removed and ERC was performed to assess the persistence of stricture. The stent was replaced if stricture remained. The duct was left stent-free in cases with improvement of the stricture (Fig. 1c). ERC was performed if any signs of stent occlusion were seen prior to the scheduled session. Technical endoscopic success is defined as successful placement of biliary stents. Stent migration was defined as the movement of the stent to a site other than across the biliary stricture. Stent occlusion was defined by ERC when the stent across the biliary stricture was suspicious in the presence of jaundice, fever, abnormal biliary enzymes and abnormal biliary findings on abdominal imaging. The period of stent patency was defined as the time interval between insertion and stent migration, occlusion or scheduled exchange of the stents. Adverse events were graded according to the system developed by Cotton et al. [17].

### Statistical analysis

For quantitative variables, we used the Mann-Whitney U test. Stent patency periods were analyzed using the Kaplan-Meier method. *P*-values < .05 were considered to be significant, if necessary P-value corrections were administered. Statistical analyses were performed using JMP (Version 8; SAS Institute Japan, Tokyo, Japan).

## Results

In this study, we analyzed a total of 98 ERC sessions for 16 patients undergoing endoscopic treatment of biliary stricture after LDLT with duct-to-duct biliary reconstruction. The most common indication for LDLT was liver cirrhosis secondary to chronic hepatitis C virus infection. The shape of the stricture site was non-visualization of the proximal duct type in 2 cases, separate type in 6 cases, narrow stricture type in 7 cases, and wide stricture type in 1 case. The mean follow-up period from/after initial endoscopic treatment was 81 months (range, 16–127 months). Information of each case was presented in Table 1.

**Table 1:** Outcomes of patients treated for biliary strictures after LDLT (*N* = 16)

Patient^a^ (age/sex)	Indications For transplant	Graft	Stricture morphology	Tip shape of the distal duct	ERCP (No)	Endoscopic Treatment Success	Number of strictures	Inside Stent (times)	Outside Stent (times)	Stent indwelling period (months)	Follow Up period (months)	Present status
30–39/sex 1	Fulminant liver failure	Left	Non-visualization	Round	1	No	1	0	0	0	108	Choledochojejunostomy
30–39/sex 2	Fulminant liver failure	Left	Wide	Tapered	14	Yes	1	11	1	105	105	Inside stent
50–59/sex 2	LC-PBC	Left	Separate	Round	8	Yes	1	8	0	85	85	Inside stent
60–69/sex 2	LC-HBV	Left	Narrow	Tapered	7	Yes	1	7	0	42	56	Stricture resolution
50–59/sex 1	LC-HCV	Left	Narrow	Tapered	5	Yes	1	3	0	19	35	Stricture resolution
60–69/sex 1	LC-HCV	Left	Narrow	Tapered	5	Yes	1	3	1	23	23	Inside stent
60–69/sex 2	LC-PBC	Left	Narrow	Round	3	Yes	1	3	0	16	16	Inside stent
50–59/sex 1	LC-HBV	Right	Narrow	Tapered	7	Yes	1	5	2	58	127	Stricture resolution
40–49/sex 1	LC-HBV	Right	Non-visualization	Round	1	No	2	0	0	0	124	Choledochojejunostomy
50–59/sex 1	LC-HCV	Right	Narrow	Tapered	8	Yes	2	5	2	17	93	Stricture resolution
40–49/sex 1	LC-NASH	Right	Separate	Round	6	Yes	2	6	0	84	84	Inside stent
60–69/sex 1	LC-alcohol	Right	Separate	Tapered	5	Yes	2	3	1	70	78	Stricture resolution
50–59/sex 1	LC-alcohol	Right	Separate	Tapered	6	Yes	1	6	0	61	61	Inside stent
60–69/sex 2	LC-HCV	Right	Separate	Tapered	4	Yes	1	4	0	19	52	Stricture resolution
50–59/sex 2	LC-PBC	Right	Narrow	Tapered	6	Yes	2	3	3	27	45	Stricture resolution
60–69/sex 1	LC-HCV	Posterior	Separate	Round	12	Yes	1	12	0	87	87	Inside stent

*LDLT* Living donor liver transplantation, *LC-PBC* Liver cirrhosis secondary to primary biliary cholangitis, *HBV* Hepatitis B virus, *HCV* Hepatitis C virus, *NASH* Non-alcoholic steatohepatitis

^a^ Age and sex of the patients are not specified to anonymize the data

**Fig. 2:**
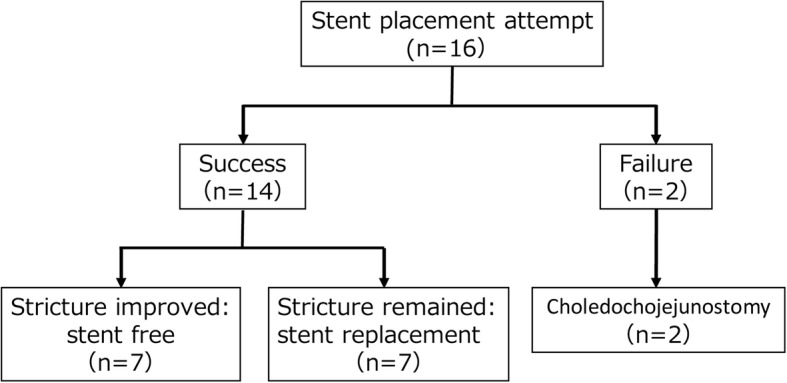
Clinical outcome of 16 patients who received stent placement against biliary strictures after LDLT

The treatment course of 16 patients is shown in Fig. 2 and Table 1. Biliary strictures were successfully treated in 14 patients (88%). Biliary strictures improved in 7 patients (44%) on repeat ERC (3–7 sessions), who therefore had their stents removed. Recurrence of the stricture was not observed during the follow up period. The median follow-up period after stent removal was 33 months (range, 13–76 months). Stent replacement was performed every 6 to 12 months for the other 7 patients (44%). The procedure failed in two patients (12%) at an initial attempt: the guidewire could not pass through severe strictures thus choledochojejunostomy was performed.

**Fig. 3:**
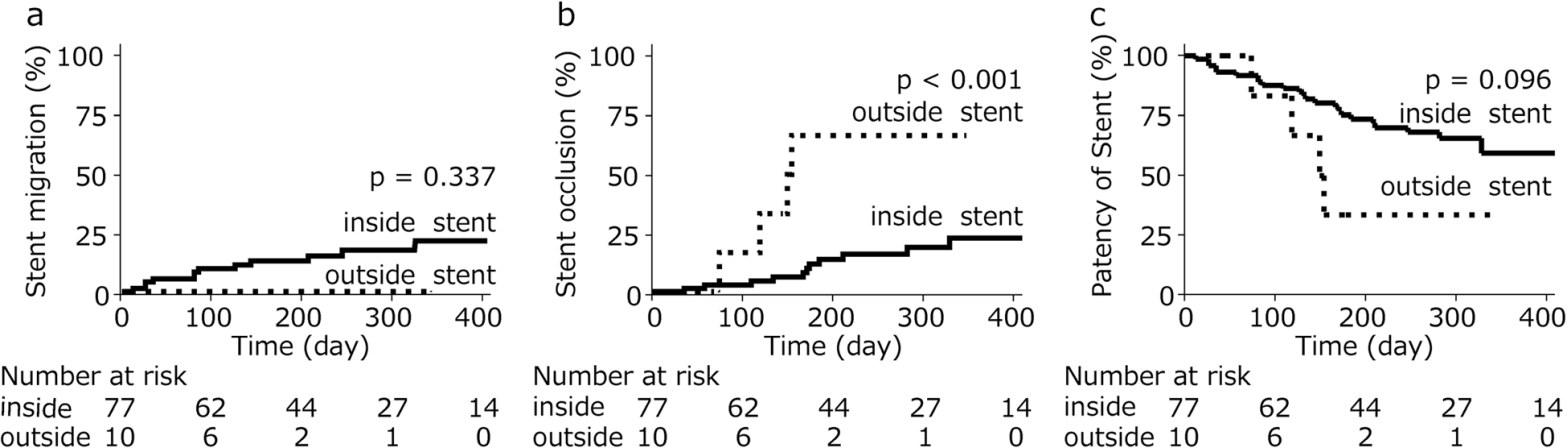
**a** Incidence of stent migration. **b** Incidence of stent occlusion. **c** Patent duration of bile duct after stent placement

Subsequently, we focused on each session. Biliary stents were placed in 87 sessions: 77 inside sessions and 10 outside sessions. Stent migration occurred in 13 of the inside stent sessions (16%), whereas none of the outside stent sessions (0%). There was no difference in stent migration between inside stent and outside stent (*P* = 0.337, Fig. 3a). The median time to stent migration was 88 days (range; 13–515) for inside stent. Stent occlusion occurred in 13 (16%) and 4 (40%) of the inside stent and the outside stent sessions, respectively. Among 17 stent occlusions, the median time to stent occlusion was 175 days (range; 36–425) for inside stent and 138 days (median; 76–158) for outside stent. The stent occlusion was significantly less in inside stent than in outside stent (*P* < 0.001, Fig. 3b). The stents were exchanged without stent occlusion in median period of 288 days (range; 96–1736) in 44 (57%) of the inside stent and 54.5 days (range; 7–356) in 6 (60%) of the outside stent. Symptoms prior to the scheduled session was observed in 17 (22%, 13 fever and 11 jaundice) of the inside stent and 3 (30%, 3 fever) of the outside stent (Table 2).

**Table 2:** Comparison of inside stent and outside stent for biliary strictures after LDLT

	Inside stent (*N* = 77)	Outside stent (*N* = 10)	*P*-value
Symptom prior to the scheduled session	17 (22%)	3 (30%)	0.575
Fever	13 (16%)	3 (30%)	0.313
Jaundice	11 (14%)	0 (0%)	0.201
Early complications	9 (12%)	1 (10%)	0.873
Pancreatitis	3 (4%)	1 (10%)	0.386
Cholangitis	6 (8%)	0 (0%)	0.360
Stone formation	14 (18%)	3 (30%)	0.400

*LDLT* Living donor liver transplantation

Median period of patency of all inside stent and outside stent were 222 (range; 8–1736) days and 99 days (range; 7–356), respectively. The patency period of the stent was longer for inside stent compared to outside stent, but not statistically different (*P* = 0.096, Fig. 3c).

Early complications were pancreatitis (6 sessions) and cholangitis (5 sessions) but all improved with conservative treatment. There were no severe adverse events. The rate of complications did not differ between the two groups (Table 2). Stone formation was observed in 14 (18%) of the inside stent and 3 (30%) of the outside stent (Table 2). Biliary stones were small and successfully removed endoscopically except one case requiring EST.

## Discussion

Endoscopic treatment of patients with LDLT may be more difficult than that of patients with DDLT [2]. Previous studies reported the success rate of endoscopic intervention in LDLT on the first attempt between 46.7 and 84.2% [15, 18, 19]. In this regard, our successful stent placing rate of 88% at the first attempt is relatively high comparing to other previous reports, presumably due to procedures carried out by board-certified specialists dedicated to biliary endoscopy with sufficient experience.

On the other hand, stricture resolution rate of endoscopic management of biliary anastomotic strictures using plastic stent was 44% (7 of 16 cases) in our study. The stricture resolution rate was not as high as the previous reports, which was achieved in 51 to 88% in LDLT cases [16, 19]. Lower rate of stricture resolution for LDLT patients is due to small caliber of intrahepatic bile duct or twisted biliary structures, likely resulted from anastomosis fibrillization and hypertrophy of the transplanted liver [16, 20]. Stricture resolution can be expected by multiple plastic stent placement and metallic stent placement [21, 22]. No multiple plastic stents or metallic stent was used, but single stent placement was used for stricture due to narrow intrahepatic bile duct in our study may be considered one of the factors contributing to insufficient stricture resolution rate and the number of endoscopic interventions (median 6 sessions). Therefore, to achieve better stricture resolution rate, we might need to change our treatment strategy, namely introduction of multiple plastic stent placement and metallic stent placement, improvement of techniques and development of novel equipment. On the other hand, recurrence rate of stricture after endoscopic treatment for post-liver transplant biliary strictures using plastic stents is 20.9% according to the meta-analysis [23]. Although median follow-up period of only 33 months after removal of plastic stent for biliary stricture, we appreciated that our careful observation and evaluation of improvement against biliary stricture led to this good outcome since there was no patient suffering from recurrence.

It has recently been reported that metallic stents are beneficial for reducing the treatment numbers and improving biliary strictures including bile duct stenosis after DDLT [24, 25]. However, the application is limited in cases of biliary stricture of LDLT because the metallic stent may block side branches and small bile duct caliber of the donor liver. Furthermore, placement of plastic stent in addition to metallic stent has been reported effective to prevent cholangitis following obstruction of the side branch duct [22]. If the large metallic stent can be indwelled safely in the biliary strictures of LDLT, it may decrease the treatment numbers. However, recurrence of biliary strictures is 17.6–20.7% even with metallic stent placement [22, 26]. Long-term plastic stent placement may be required in recurrent cases [7]. Hence it is important to clarify the patency periods of plastic stent.

In the previous reports, outside stents are usually exchanged every 2 to 4 months to minimize stent occlusion, and to prevent cholangitis or stone formation, although there is no report on the patency of the outside stent in LDLT patients [12, 27]. On the other hands, Tsujino et al. have alluded to their experience in the area with 63 patients with LDLT who underwent inside stent placement. The median interval of inside stent exchange was 161 days (5.4 months) [12]. Kurita et al. reported that the patency period of inside stent was 189 days (6.3 months) [16]. In our study, median patency period of inside stent was 222 days (7.4 months) and outside stent was 99 days (3.3 months). However, the stent was replaced before occlusion in 57% of the cases and considered as stent occlusion as such the exact period patency is unclear. Nevertheless, prevention of duodenal fluid reflux into the bile duct by preserving intact sphincter of Oddi might be important for long-term patency of inside stent.

With regards to the major issues of inside stent, stent migration occurred in 11% of biliary strictures post LDLT in the previous reports [16, 28]. Similarly, stent migration occurred in 16% in our study. The reason why there was no significant difference in the stent patency period is probably due to stent migration, although the stent occlusion was significantly lower in inside stent than in outside stent. Hence it is important to select an appropriate stent suitable for the bile duct to maximize the benefit of inside stent and to prevent stent migration.

When the patent period of the inside stent gets longer, indwelling stents can cause stent-stone complexes. As a result, endoscopic removal may be difficult and may require surgery [17, 29]. In our study, stent exchange was scheduled every 6 to 12 months and small stones were formed in some cases. However, they were removed endoscopically at the time of stent replacement. We only had a few cases in which endoscopic treatment was necessary within 6 months due to stent migration or occlusion. In addition, median patency periods of inside stent were at least 7.4 months or more. Therefore, we recommend periodic replacement of the inside stent every 6 to 12 months.

Our study has limitations. This was a non-randomized, retrospective, single center experience with a relatively small number of patients. Therefore, no difference in complications between the two groups remain unclear. Since the decision of inside stent and outside stent was based on the judgment of the operator, selection bias is a concern. A randomized prospective multicenter study with a larger patient population is needed to further evaluate the efficacy of inside stent.

## Conclusions

In summary, the endoscopic treatment of biliary strictures using an inside stent is useful. The stent occlusion was significantly less in inside stent than in outside stent. To suppress the stent-stone complex, we recommend periodic replacement of the inside stent every 6 to 12 months.

## Data Availability

The datasets used and/or analysed during the current study are available from the corresponding author on reasonable request.

## Abbreviations

CBD: Common bile duct
CT: Computed tomography
DDLT: Deceased donor liver transplant
ERCP: Endoscopic retrograde cholangiopancreatography
LDLT: Living donor liver transplantation
EST: Endoscopic sphincterotomy
